# Comparison of the Efficacy of Oral *versus* Intravascular Magnesium in the Prevention of Hypomagnesemia and Arrhythmia after CABG

**DOI:** 10.21470/1678-9741-2018-0070

**Published:** 2018

**Authors:** Mansour Jannati, Shahrbanoo Shahbazi, Laleh Eshaghi

**Affiliations:** 1 Cardiovascular Surgery Ward, Faghihi Hospital, Shiraz University of Medical Sciences, Shiraz, Iran.; 2 Department of Anesthesiology, Shiraz Anesthesiology and Critical and Care Research Center, Nemazee Hospital, Shiraz University of Medical Sciences, Shiraz, Iran.; 3 Department of Anesthesiology, Shiraz University of Medical Sciences, Shiraz, Iran.

**Keywords:** Coronary Artery Bypass, Magnesium Sulfate, Magnesium Hydroxide, Administration, Intravenous, Administration, Oral, Arrhythmias, Cardiac

## Abstract

**Objective:**

Cardiac arrhythmias are a common challenge following open-heart surgeries. Hypomagnesemia is believed to be correlated with this condition. Prophylactic intravenous magnesium supplementation has been practiced for a long time in patients undergoing CABG. This study was designed in an attempt to compare the efficacy of oral *versus* intravenous routes in the prevention of hypomagnesemia and arrhythmia.

**Methods:**

In this interventional clinical study, 82 patients were randomly assigned to 2 groups. All patients were evaluated for baseline serum magnesium level and arrhythmias. One group received 1,600 mg of oral magnesium hydroxide through nasogastric (NG) tube prior to surgery, while the other group received 2 g of magnesium sulfate during the induction of anesthesia. The serum magnesium level was monitored for 48 hours after the operation. The difference in preoperative hypomagnesemia was non-significant (Sig: 0.576).

**Results:**

During the operation, the serum magnesium level peaked around 4 mg/dL, and no hypomagnesemia was detected in any patient. Although the curve of oral group declined parallel and below that of intravenous (IV) group, no significant differences were detected during postoperative monitoring. In addition, a prevalence of arrhythmia of 13.9% and 6.5% was noticed in IV and oral groups, respectively (OR: 0.428).

**Conclusion:**

Providing 1,600 mg of oral magnesium supplement to patients is as effective as 2,000 mg of magnesium sulfate IV in preventing hypomagnesemia and arrhythmia after CABG. Thus, the authors introduce this treatment regimen as a promising and cost-effective method.

**Table:** 

Abbreviations, acronyms & symbols	
AF	= Atrial fibrillation
CABG	= Coronary artery bypass graft
CAD	= Coronary artery disease
CPB	= Cardiopulmonary bypass
ECG	= Electrocardiogram
IABP	= Intra-aortic balloon pump
ICU	= Intensive care unit
IV	= Intravenous
NG	= Nasogastric
SUMS	= Shiraz University of Medical Sciences

## INTRODUCTION

Patients undergoing open-heart surgeries, such as coronary artery bypass graft (CABG), are prone to develop new-onset postoperative cardiac arrhythmias, particularly atrial fibrillation (AF), with an incidence of approximately 20 to 50%^[1]^. Despite the overall advances in surgical and anesthetic procedures, the trend has not shown any decrease over the past years^[2]^. This condition usually occurs within 24 to 96 hours after surgery^[3]^ and, due to its benign and self-limiting course, no long-term sequelae are expected^[2]^. However, the burden of associated complications cannot be overlooked. AF predisposes patients to hemodynamic disturbance as a result of reduced cardiac output and elevated atrial and ventricular end-diastolic pressures^[4]^. Other complications include thromboembolism, stroke and palpitations, which eventually lead to prolonged hospital stay and increased costs^[2]^. Thus, these adverse clinical and financial outcomes justify the excessive efforts made to improve preventive measures and management guidelines.

There are numerous risk factors attributed to postoperative cardiac arrhythmia. Hypomagnesemia has long been regarded as having a well-documented contributory role. Supporting evidence arises from reports of arrhythmias in patients with low serum magnesium levels (less than 1.5 mEq/L) and suppression of arrhythmias through magnesium replacement^[4]^. Patients with severe coronary artery disease are more likely to have magnesium deficiency. In addition, cardiac surgery further exacerbates this disadvantage, in which a decline is often detected on the first day after CABG, before a gradual rise to preoperative levels by the fourth day^[4]^. Therefore, prophylactic magnesium therapy is a common practice aimed at alleviating this undesirable condition.

The present study pursues a twofold purpose; one is evaluating the efficacy of oral *versus* intravascular magnesium administration in preventing postoperative hypomagnesemia in patients undergoing CABG surgery and the second goal is examining the potential of oral supplementation to prevent arrhythmias. To the best of our knowledge, there is no similar experiment in the literature comparing oral and intravascular routes of administration.

## METHODS

This interventional clinical study was conducted on 82 patients with coronary artery disease (CAD), who were scheduled for CABG operation in hospitals affiliated with Shiraz University of Medical Sciences (SUMS), Shiraz, Iran, during the year 2015. Exclusion criteria consisted of history of renal diseases, liver diseases, arrhythmias non-responsive to medical treatment and hypersensitivity to magnesium. In addition, consumption of magnesium-containing medications, antiarrhythmic drugs, beta-blockers, calcium channel blockers, and diuretics was regarded as another exclusion criterion. Additionally, patients who underwent exploratory surgery after CABG operation due to bleeding, or had intra-aortic balloon pump (IABP) insertion were eventually omitted from the study. The Ethics Committee of SUMS approved all the processes in this study.

Prior to surgery, each patient was fully instructed on the method of the study and was required to fill out an informed written consent. Patients were equally randomized to the oral or intravenous (IV) group, using the blocking method. Subsequently, baseline magnesium level and electrocardiographic study were obtained. All operations were performed by the same cardiac surgeon and both patients and medical staff were blinded about the groupings. The sample size was calculated in at least 36 subjects for each group (µ1- µ2 = 0.02, ϭ = 0.03, 1-β = 80%) and a *P*-value <0.05 was considered significant.

One group received 1,600 mg oral magnesium hydroxide (400 mg = 5 cc) through nasogastric (NG) tube before the operation, while the other group received 2 g of magnesium sulfate IV during the induction of anesthesia. General anesthesia was administered with 0.1-0.2 mg/kg midazolam, 0.7-1 mg/kg sufentanil, 1-2 mg/kg sodium thiopental and 0.2 mg/kg pancuronium. Anesthesia was maintained by propofol during the surgery. Blood samples were immediately obtained, for a second time, after induction of anesthesia. After sternotomy, the graft (internal thoracic artery, saphenous vein or radial artery) was resected. Cannulation was made by the surgeon and the patient was connected to the cardiopulmonary pump. The aorta was clamped and cardioplegia solution was injected through the cannula. This solution contains 16 g of magnesium. Meanwhile, the patient was cooled down to 34°C. After anastomosis, the patient was warmed up again. At this time, another serum sample was collected. Afterwards, 2 g of magnesium bolus was administered for both groups through cardiopulmonary pump. In case of occurrence of arrhythmia after declamping, types and treatments were recorded. Finally, the proximal anastomosis was performed. After chest tube insertion and a thorough emulation for bleeding, the sternum was sutured. Three other serum samples were collected when the patients were transferred to intensive care unit (ICU), and 12 and 24 hours after transfer. Magnesium levels below 2 mg/dL are considered as hypomagnesemia. Data were analyzed by IBM SPSS Statistics using independent T-test and chi-square test. The results are demonstrated descriptively and analytically.

## RESULTS

Initially 82 patients were involved in this study; however, 15 were subsequently omitted due to exploratory surgery for bleeding or IABP insertion. The remainder were 31 (53%) patients in IV group and 36 (46%) in the oral group (Table 1).

**Table 1:** Demographic and perioperative data in both IV and oral groups.

	IV Mg	Oral Mg	*P*-value (Sig. <0.05)
Number	31	36	-
Age	64.11±11.518	63.06±9.609	0.179
Sex (male)	71.4%	72.2%	0.938
Sex (female)	28.6%	27.8%	0.938
Weight (kg)	67.83±11.175	65±11.578	0.729
Cardiopulmonary bypass duration (min)	77.72±14.65	82.55±13.66	0.131
Cross-clamp duration (min)	42.61±11.27	45.61±9.99	0.329
Cardioplegia volume (mL)	1353.61±344	1337.10±227	0.909
Hypomagnesemia	2.8%	-	1
Preoperative arrhythmia	11.9%	5.6%	0.442
ICU stay (hour)	109.33±110.533	102.97±122.053	0.498

ICU=intensive care unit

**Fig. 1:**
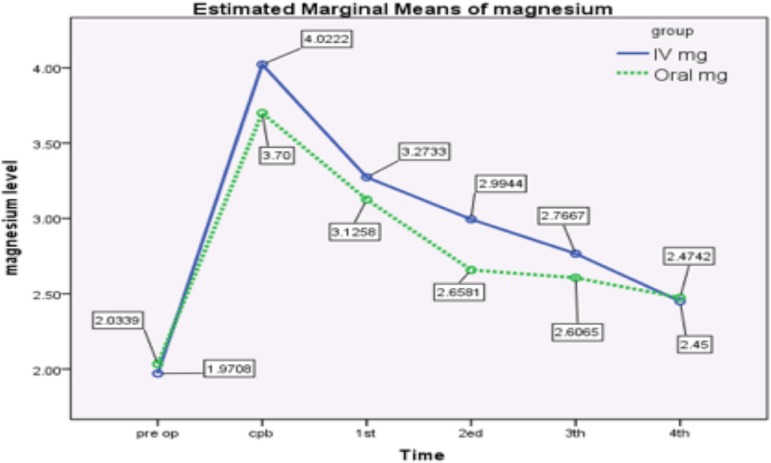
Serum magnesium level of both groups up to 48 hours after the operation. CPB=cardiopulmonary bypass, IV=intravenous; Pre op=preoperative period

The serum magnesium level of both groups up to 48 hours after the operation is shown in the chart below (Figure 1).

The original magnesium level in the orally treated group was 2.0339±0.7772 mg/dL, compared to the magnesium level in the parenteral group; 1.9708±0.6678 mg/dL. The difference in preoperative hypomagnesemia was non-significant in both groups (*P*-value =0.576) (Table 2). During the operation, the curve rises to about 3.7 to 4 mg/dL, which is predictable with regard to perioperative administration of magnesium. No hypomagnesemia was detected in the groups during the operation and cardiopulmonary bypass (CPB). Afterwards, it gradually declines, but with a faster inclination during the first 12 hours. During this period, the magnesium level of oral group is lower, but parallel to IV group. Generally, patients in both groups presented hypomagnesemia without significant differences.

**Table 2:** Prevalence of hypomagnesemia before, during and after the operation in both IV and oral groups.

	IV (n=31)	Oral (n=36)	*P*-value
Preoperative	16.7%	22.2%	0.576
During CBP	-	-	-
Postop (0 hour)	2.8%	-	1
Postop (12 h)	-	3.2%	0.463
Postop (24 h)	-	3.2%	0.463
Postop (48 h)	2.8%	-	1

CPB=cardiopulmonary bypass

**Fig. 2:**
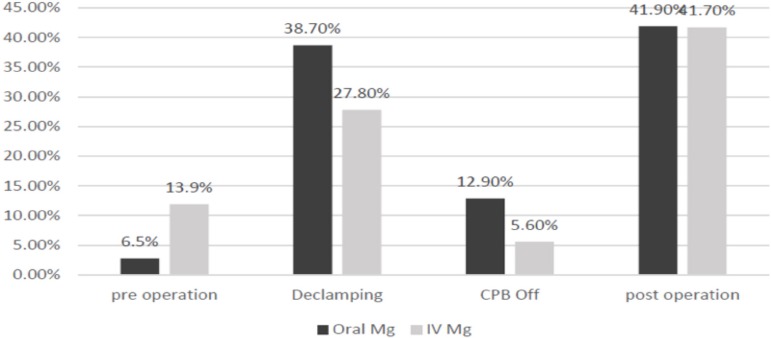
Prevalence of arrhythmia in oral and IV groups. CPB=cardiopulmonary bypass, IV=intravenous

In addition, a prevalence of preoperative arrhythmia of 13.9% and 6.5% was observed in IV and oral groups, respectively (OR: 0.428). However, during the operation, patients in oral group have the advantage, until the postoperative period, when both presented the same prevalence (Figure 2).

**Fig. 3:**
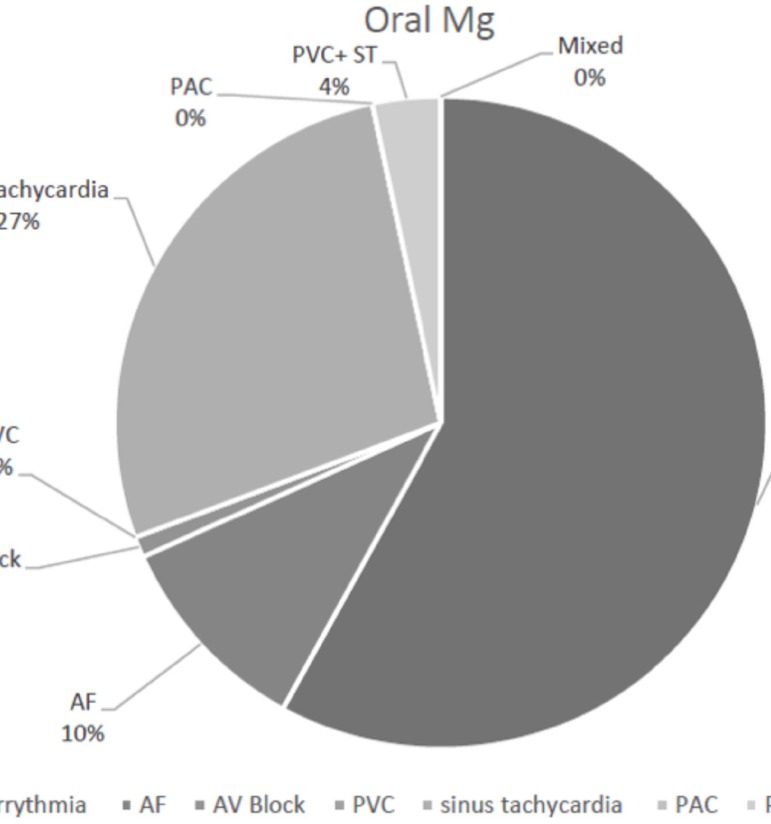
Arrhythmia in oral group. AF=atrial fibrillation; AV Block=atrioventricular node block; PAC=premature atrial contraction; PVC=premature ventricular contraction; ST=sinus tachycardia

**Fig. 4:**
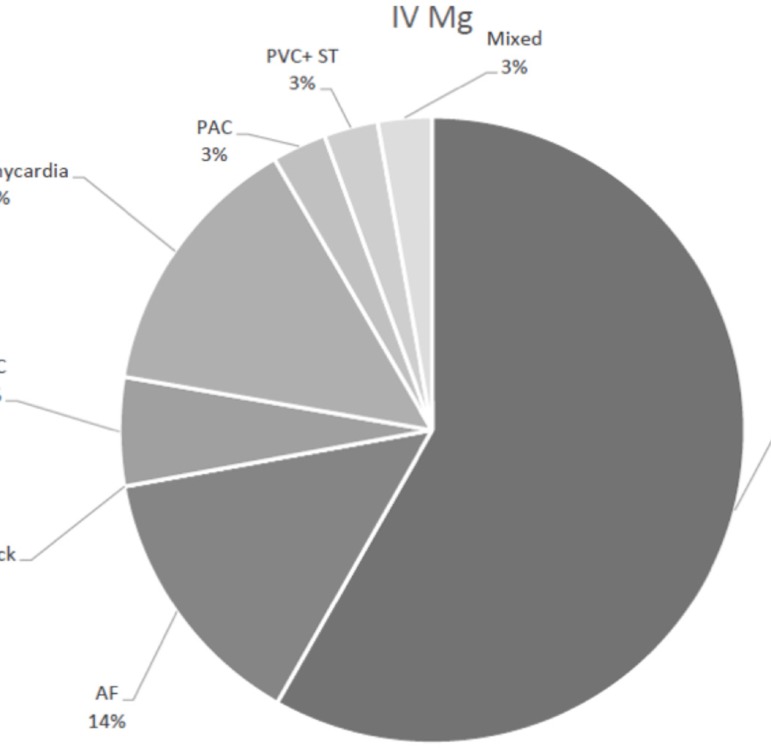
Arrhythmia in IV group. AF=atrial fibrillation; AV Block=atrioventricular node block; PAC=premature atrial contraction; PVC=premature ventricular contraction; ST=sinus tachycardia

The percentages of different types of arrhythmias are described in the Figures 3 and 4.

## DISCUSSION

Magnesium is a bivalent cation essential for the maintenance of physiological transmembrane gradients of sodium and potassium ions^[5]^. It acts as a cofactor of the energy source for contractility, adenosine-triphosphatase, and protects the myocardium against ischemic reperfusion injury^[6,7]^. Serum magnesium concentration decreases after cardiac surgery, which is usually normalized after 3 to 5 days^[8]^.

This deficiency leads to cardiovascular clinical manifestations such as hypertension and electrocardiogram (ECG) changes, as well as supraventricular and ventricular arrhythmias^[8]^. Several mechanisms have been proposed regarding the role of magnesium deficiency in postoperative AF, but no clear explanation has been fully agreed upon. Some refer to hemodilution, extracellular to intracellular shift, use of intraoperative diuretics, increased urinary loss and catecholamine discharge, and high level of epinephrine^[3,4,9]^.

Magnesium treatment has been shown to decrease the incidence of cardiac arrhythmias by prolongation of the refractory period^[10,11]^. Despite the extensive investigation, a great lack of consistency is seen in the literature regarding prophylactic magnesium therapy. In addition, indications, dosage and time should be clarified. Conventional methods involve systemic and supplementation of cardioplegic solution^[6]^. The new point of the present study is the application of oral route for the first time and similar previous experiments are nonexistent. In addition, as the results indicated, preoperative oral magnesium loading has a potential promising efficacy.

Several studies strongly encourage the preoperative loading of magnesium as a suitable alternative agent in the prevention of postoperative arrhythmia, observing its low cost and safety^[2,12-16]^. This is in accordance with the recommendations of European and Canadian guidelines^[17,18]^. However, this is contrary to the recent US guideline, which did not mention magnesium as a choice of routine management, along with other studies, which report only minimal benefit or lack of any particular advantages^[19-22]^. Overall, it seems that variety in design and quality of studies can be responsible for the conflicting results. Nonetheless, the findings of the present study indicate that in the settings mentioned in the study, oral and IV magnesium can be of equal value.

### Limitation

Limitations and suggestions: studies with larger samples and use of a control group.

## CONCLUSION

Providing patients with 1,600 mg oral magnesium supplement is as effective as 2,000 mg of magnesium sulfate IV in preventing hypomagnesemia and arrhythmia after CABG. Thus, authors suggest that this treatment regimen is promising.

**Table:** 

Authors' roles & responsibilities	
MJ	Substantial contributions to the conception or design of the work; or the acquisition, analysis, or interpretation of data for the work; agreement to be accountable for all aspects of the work in ensuring that questions related to the accuracy or integrity of any part of the work are appropriately investigated and resolved; final approval of the version to be published
SS	Drafting the work or revising it critically for important intellectual content; agreement to be accountable for all aspects of the work in ensuring that questions related to the accuracy or integrity of any part of the work are appropriately investigated and resolved; final approval of the version to be published
LE	Agreement to be accountable for all aspects of the work in ensuring that questions related to the accuracy or integrity of any part of the work are appropriately investigated and resolved; final approval of the version to be published
